# Genetic structure of the endemic *Dipterocarpus condorensis* revealed by microsatellite markers

**DOI:** 10.1093/aobpla/plac007

**Published:** 2022-02-23

**Authors:** Duc Minh Nguyen, Hong Lan Phan Nguyen, Tam Minh Nguyen

**Affiliations:** 1 Institute of Genome Research, Vietnam Academy of Science and Technology, 18 Hoang Quoc Viet, Cau Giay, Hanoi 100000, Vietnam; 2 Faculty of Biotechnology, Graduate University of Science and Technology, Vietnam Academy of Science and Technology, 18 Hoang Quoc Viet, Cau Giay, Hanoi 100000, Vietnam; 3 Institute of Ecology and Biological Resources, Vietnam Academy of Science and Technology, 18 Hoang Quoc Viet, Cau Giay, Hanoi 100000, Vietnam; 4 Vietnam National Museum of Nature, Vietnam Academy of Science and Technology, 18 Hoang Quoc Viet, Cau Giay, Hanoi 100000, Vietnam

**Keywords:** Admixture, bottlenecks, conservation genetics, dipterocarp, fragmentation

## Abstract

Anthropogenic disturbances in tropical forests often affect the genetic diversity of a species. *Dipterocarpus condorensis* is an endangered species in the tropical forests of south-eastern Vietnam, both from its over-exploitation and habitat loss. Therefore, knowledge of population genetic diversity and population structure is essential for identifying the species conservation measures. In the present study, we evaluated genetic diversity and population structure using nine microsatellites for 183 individual trees from eight populations, representing the distribution range of *D. condorensis* in Vietnam. Two clustering approaches (Bayesian analysis and discriminant analysis of principal components) revealed that all studied individuals clustered into three genetic groups, which were related to gene flow across the range of *D. condorensis* in the lowland tropical forests of south-eastern Vietnam. Limited gene flow was implicated in anthropogenic disturbance. Genetic differentiation among populations was relatively low (the Weir and Cockerham index of 0.122 and the Hedrick index of 0.149) and showed significant differentiation. The genetic variability of the populations was low (*H*_O_ = 0.298 and *H*_E_ = 0.324), which suggested the negative effects of habitat degradation and over-exploitation. Our studies also determined that *D. condorensis* populations can have undergone recent bottlenecks. We recommend conservation activities for this species based on these results.

## Teaser

*Dipterocarpus condorensis*is an endangered species in forests of south-eastern Vietnam, both from its over-exploitation and habitat loss. In this study, we evaluated genetic diversity and population structure for eight populations, representing the distribution range of the species. Bayesian analysis and discriminant analysis of principal components revealed three genetic groups, which were related to gene flow among different areas. Limited gene flow was implicated in anthropogenic disturbance. Genetic differentiation was relatively low and showed significant differentiation. The genetic variability of populations was low, which suggested the negative effects of habitat degradation and over-exploitation. Populations may have undergone recent bottlenecks.

## Introduction

Habitat degradation and fragmentation are major threats to many plant species, which affect species distribution, population size and genetic variability (Young *et al.* 1996). Small populations often have negative genetic consequences such as inbreeding depression, increased genetic drift and bottlenecks due to reduced population sizes and increased isolation between populations (Kiviniemi 2008; Barmosky *et al.* 2011), leading to a reduction in fitness and an increase of susceptibility to environmental stochasticity with respect to loss of genetic variability of the populations and the fixation of potentially deleterious alleles (Hedrick and Kalinowski 2000; O’Grady *et al.* 2006; Gargano *et al.* 2015). The decline in genetic variability is associated with decreased habitat availability (Helm *et al.* 2009). In addition, gene flow may be restricted by the formation of isolated small populations (Ellstrand and Elam 1993). The adaptive and evolutionary potential of a species in nature is influenced by its degree of genetic variability and differentiation. Understanding the amount of genetic variability in a population can provide the knowledge to develop effective strategies for species conservation and to ensure the long-term viability of a species (Hedrick and Miller 1992). Maintaining high genetic variability is one of the key requirements for preserving population survival (Hoban *et al.* 2020). Endangered plant species are often characterized by small population sizes and fragmented habitats, which are facing environmental and biological stresses compared to other species (Frankham *et al.* 2002; Aguilar *et al.* 2008).

Dipterocarps are the predominant forest species and play a crucial role in both the ecology and economics of tropical forests in Vietnam. More than 45 dipterocarp species in six genera have been identified, the majority of which are native and endemic (Nghia 2005). Due to their economic value and local timber demand, dipterocarps are over-exploited. Furthermore, in recent years, increasing human pressure and rapid economic development have resulted in a significant reduction in forest areas and a rise in the degree of fragmentation of the surviving forests. These trends are having a negative impact on the dipterocarp habitats. These changes have seriously threatened the survival of dipterocarps. Currently, 33 dipterocarp species are at threat in the global level. Dipterocarp species *Dipterocarpus condorensis* has been commonly used in making furnitures and building. The species is used as a valuable source of essential oils. It is also widely exploited due to its commercial trade. The dipterocarp can be found in the lowland rainforests of Vietnam. This species is bisexual and pollinated by insects. The flowers are large, actinomorphic and scented. Fruiting appears only once in every 2–3 years, with fruit maturation taking place between the months of April and May. A single-seeded nut with a wing-like calyx makes up the fruit. Seeds are dispersed by wind. *Dipterocarpus condorensis* is only found in forests on ancient alluvial rocks, granite and basalt rocks along coast provinces of the southern central region from Ba Ria–Vung Tau to Phu Yen. Deforestation, fragmentation and unsustainable management activities such as selective logging have all been detrimental to *D. condorensis* habitats caused due to the activities of local people and forestry enterprises exploiting the species for its valuable timber and resin. Logging leads to fragmented habitats and low population density. This threatens the long-term survival of species resources. Globally, *D. condorensis* is classified as CR A1cd, B1 + 2c (IUCN 2021). This species has been listed as Endangered in Vietnam due to habitat loss and over-exploitation (BKHCN 2007).

Information on ecology and genetic diversity within and among populations is required for species conservation and management efforts. Powerful biological techniques are needed to obtain such information, especially a better understanding of genetic processes. Microsatellite (SSR) markers are powerful tools because of their high polymorphism and codominant inheritance. Various SSR markers have been developed for dipterocarps (Terauchi 1994; Ujino *et al.* 1998; Isagi *et al.* 2002). In studies of genetic diversity within and among dipterocarp populations, these markers have been widely used (Pandey and Geburek 2009; Muhammad *et al.* 2016). To date, genetic research on *D. condorensis* has been limited. However, previous studies have shown high genetic variability in some dipterocarp species, such as *Shorea leprosula* (Ng *et al.* 2004), *S. robusta* (Pandey and Geburek 2009), *Dryobalanops aromatica* (Harada *et al.* 2018) and *Dipterocarpus dyeri* (Tam *et al.* 2020), reflecting the life history traits and mating system. Several threatened dipterocarps have low genetic variability, such as *Parashorea malaanonan* (Abasolo *et al.* 2009), *Dipterocarpus alatus* (Tam *et al.* 2014) and *Hopea odorata* (Trang *et al.* 2014) suggesting the loss and fragmentation of habitats.

In the present study, we applied population genetic analysis of *D. condorensis* in the tropical forests of south-eastern Vietnam to evaluate the degree of genetic variability within and among the populations, and to determine the genetic structure for all studied individuals from the eight populations. The outputs of the study are aimed towards providing guidelines for the conservation, management and restoration of this species.

## Materials and Methods

### Sample collection and DNA isolation

We collected samples at eight locations: four in the Ba Ria–Vung Tau province, three in Binh Thuan and one in Khanh Hoa **[see****Supporting Information—Table S1****and****Fig. S1****]**. Human activities, such as agricultural expansion and over-exploitation of forest trees for trade, firewood collection and building purposes in the 1980s and 1990s, have had a significant impact on the original vegetation at these sites. However, a three-storey structure of vegetation was also found in these studied areas. The spatial distribution and age-class structure of forest trees are altered in degraded habitats. As a result, the spatial distribution and age-class composition of trees at these locations changed. These areas consist of ancient alluvial rocks, granite and basalt rocks, with low relief and gentle slopes, where water levels rise rapidly in both dry and rainy seasons, according to geomorphology. This species is found to be disturbed in the forest patches in its respective protected area due to selective logging. The clearance for agriculture and human settlements has dispersed all of the *D. condorensis* populations in each nature reserve.

*Dipterocarpus condorensis* is a large tree, in this study we randomly sampled the inner bark of 183 adult trees from eight populations across the entire contribution sites, representative of the natural distribution range of the species. To avoid sampling clonal material, we kept the distance between collected trees within the population to at least 50 m. We recorded each collection site using a global position system (GPS) receiver. Samples were packed in plastic bags with silica gel in the field and then transferred to the Laboratory Molecular Biology, Institute of Genome Research and stored at −45 °C until DNA extraction.

We extracted genomic DNA from the samples using the modified CTAB method proposed by Doyle and Doyle (1990). Liquid nitrogen was added to about 100 mg per each sample before it was ground in Mixer mill MM 400 (Retsch). The quality and quality of DNA samples were checked by electrophoresis on 1 % agarose gel, as well as by a spectrophotometer using the NanoDrop 2000C (Thermo Scientific, Wilmington, Delaware, USA).

### Microsatellite amplification

The polymerase chain reaction (PCR) was performed in a 25-µL solution volume containing 17.5 µL of ddH_2_O, 2.5 µL of 1× PCR buffer (10 mM Tris-HCl, 50 mM KCl, pH 8.4), 1.0 µL of MgCl_2_ (2.5 mM), 0.5 µL of dNTPs (0.2 mM each), 1.5 µL of genomic DNA (10 ng), 1.0 µL of *Taq* DNA polymerase at 1.25 units and 0.5 µL of each primer at 10 pmol. The SSR primer pairs were analysed by testing seven primer pairs for *Shorea curtisii* (Ujino *et al.* 1998), four for *Dipterocarpus tempehes* (Isagi *et al.* 2002) and five for *Dryobalanops lanceolata* (Terauchi 1994). A total of nine SSR primers were chosen for this analysis because they generated clear and reproducible profiles **[see****Supporting Information—Table S2****]**. The reaction mixture was subjected to amplification in the GeneAmp PCR System 9700 (Applied Biosystems), under the following thermal cycles: an initial denaturing step at 94 °C for 2 min, followed by 40 cycles consisting of 1 min at 94 °C, 30 s annealing temperature for each primer pair at 54–56 °C and 1 min extension at 72 °C, and 10 min final at 72 °C to complete the extension of any remaining products before holding the samples at 4 °C until they were analysed. The amplification products were separated using a Sequi-Gen®GT DNA electrophoresis system of 6 % polyacrylamide gels in 1× TAE buffer and then visualized by GelRed™ Nucleic Acid Gel Stain. Allele sizes were detected by the Gel-Analyzer software of GenoSens1850 (Clinx Science Instruments Co., Ltd) with a 50-bp DNA ladder (Invitrogen) **[see****Supporting Information—Table S3****]**.

### Data analysis

#### Genetic diversity.

 Null alleles and other genotyping errors were detected using the MICRO-CHECKER v. 2.0 software (van Oosterhout *et al.* 2004), with 1000 bootstrap iterations over loci to generate the expected homozygote and heterozygote frequencies. We used the CERCUS program (Kalinowski *et al.* 2007) to estimate the PIC (polymorphism information content) value for each locus. Genetic diversity was estimated based on the SSR allele frequencies, including the number of alleles (*N*_A_), number of effective alleles (*N*_E_) per locus, number of private alleles (*N*_P_), observed (*H*_O_) and expected (*H*_E_) heterozygosities across loci and populations, proportion of polymorphic loci (PPL) and gene flow (*N*_m_) using GenAlEx v. 6.5 (Peakall and Smouse 2012). We calculated the gene flow between populations using the *F*_ST_ value, *N*_m_ = (1/*F*_ST_ − 1)/4. In addition, the fixation index (*F*_IS_—inbreeding coefficient) was calculated for each locus and population by FSTAT v. 2.9.3 (Goudet 2001). The *F*_IS_ values were corrected for null allele frequencies based on the individual inbreeding model (IIM) using INEst (Chybicki and Burczyk 2009). Tests for deviation from the Hardy–Weinberg equilibrium at each locus and the linkage disequilibrium for each locus pairwise combination in each population were performed by GENEPOP’007 (Rousset 2008). Testing for deviations in heterozygosity from expected heterozygosity under mutation–drift balance for each population was performed using BOTTLENECK v. 1.2 (Piry *et al.* 1999). A two-phase model was used and tested via the Wilcoxon signed-rank test. The proportion of the stepwise mutation model was set to 70 % under default settings.

#### Genetic differentiation and population structure.

We calculated the genetic differentiation between populations according to *F*_ST_ (Weir and Cockerham 1984) and *G*ʹ_ST_ values (Hedrick 2005) using GenAlEx. The significance of the *F*_ST_ values in population pairs across all loci was tested at the 0.05 significance level using ARLEQUIN v. 3.1 (Excoffier *et al.* 2005). The significance testing for the variance components in the analysis of molecular variance (AMOVA) was performed on the basis of 10 000 permutations using ARLEQUIN v. 3.1. We further analysed population genetic structure using different approaches. Bayesian clustering was implemented to analyse population structure using STRUCTURE v. 2.3.4 (Pritchard *et al.* 2000). Setting the admixture model with correlated allele frequencies, 10 separate runs of the number of groups in the data set (*K*) were implemented for *K* between 1 and 10 at 500 000 Markov Chain Monte Carlo (MCMC) repetitions and at 100 000 burn-in period. To determine the optimal value of *K*, we used STRUCTURE HARVESTER (Earl and von-Holdt 2012) to detect the number of groups that best fit the data set based on the ∆*K* by Evanno *et al.* (2005). After the best *K* value was inferred, the replicated results were aligned using CLUMPP v. 1.1.2 (Jakobsson and Rosenberg 2007) and bar plots of the assigned cluster membership were drawn using DISTRUCT v. 1.1 (Rosenberg 2004). We also conducted discriminant analysis of principal components (DAPC) using the *adegenet* package for the R v. 4.0.2 software to identify clusters of genetically related individuals (Jombart *et al.* 2010). Discriminant analysis of principal components was implemented without prior information on population origin. The number of clusters (*K*) was performed from 1 to 20, and the ‘optimal’ clusters were determined based on the Bayesian information criterion. The DAPC was also performed the prior information to analyse for assignment of individuals to populations. The *complot* function in *adegenet* was implemented to visualize the genetic clusters. Fourteen first principal components (PCs) (98.5 % of conserved variance) of PCA and seven discriminant eigenvalues were retained using the *xval*DAPC function.

## Results

### Microsatellite polymorphism

All nine SSR markers produced a total of 26 different alleles ranging in size from 122 to 301 bp, across 183 trees of the eight natural populations of *D. condorensis*. Of these alleles, two alleles were private. The nine SSR loci were polymorphic in *D. condorensis* (Table 1). The number of alleles per locus (*N*_A_) ranged from 2 at two loci to 4 at one locus, an average of 2.9. The effective allele (*A*_E_) ranged from 1.2 at two loci to 2.1 at one locus, an average of 1.6. Polymorphism information content values ranged from 0.159 at the dipt1 locus to 0.509 at the dipt2 locus, an average of 0.33. Eight of the nine loci displayed heterozygote deficits under the Hardy–Weinberg equilibrium that indicated inbreeding or the presence of null alleles. Micro-Checker analysis detected null allele frequencies at seven loci (*P* < 0.05). The observed (*H*_O_) and expected (*H*_E_) heterozygosity averaged 0.296, ranging from 0.157 at dipt1 to 0.465 at dipt3 and 0.324, ranging from 0.162 at dipt1 to 0.477 at dipt2, respectively. The average fixation index (*F*_IS_) over all populations for each locus was 0.071. The studied nine loci had a positive fixation index, indicating an excess of homozygotes and inbreeding. However, among these loci, two loci had significant inbreeding (*P* < 0.05). The inbreeding coefficient determined for the total populations (*F*_IT_) averaged 0.183 (Table 1), ranging from 0.07 (dipt4 and dipt6) to 0.375 (dipt7), suggesting an excess of homozygosity in the populations.

**Table 1.** Genetic parameters in nine loci for *D. condorensis.*

Locus	*N*_A_	*A*_E_	*N*_P_	PIC	*H*_O_	*H*_E_	*F*_IS_	Null allele	*F*_IT_	*F*_ST_	*G*’_ST_	HW	*N*_m_
dipt1	3	1.2	1	0.159	0.157	0.162	0.03	no	0.091	0.063	0.02	nd	3.736
dipt2	3	2.1	—	0.509	0.412	0.477	0.136*	0.144	0.29	0.178	0.305	***	1.155
dipt3	3	2	—	0.482	0.465	0.47	0.01	0.085	0.181	0.173	0.289	ns	1.194
dipt4	2	1.2	—	0.169	0.176	0.177	0.004*	0.071	0.07	0.066	0.04	nd	3.532
dipt5	3	1.3	1	0.204	0.185	0.202	0.083	0.077	0.188	0.114	0.1	nd	1.94
dipt6	4	1.4	—	0.258	0.254	0.256	0.009	No	0.07	0.062	0.031	nd	3.806
dipt7	3	1.7	—	0.429	0.309	0.351	0.118	0.176	0.375	0.291	0.432	***	0.609
dipt8	3	1.8	—	0.423	0.379	0.445	0.15	0.08	0.189	0.046	0.012	ns	5.132
dipt9	2	1.6	—	0.336	0.343	0.382	0.102	0.09	0.193	0.102	0.112	ns	2.199
Mean	2.9	1.6		0.33	0.296 (0.021)	0.324 (0.022)	0.071 (0.02)		0.183 (0.034)	0.122 (0.027)	0.149 (0.051)		2.589 (0.509)

*N*_A_ = number of alleles; *A*_E_ = effective alleles; *N*_P_ = number of private alleles; PIC = polymorphism information content; *H*_O_ and *H*_E_ = observed and expected heterozygosity; *F*_IS_ = fixation index; Null allele = the average null allele frequency; *F*_IT_ = coefficient of total inbreeding; *F*_ST_ = genetic differentiation index of Weir and Cockerham (1984); *Gʹ*_ST_ = genetic differentiation index of Hedrick (2005); *N*_m_ = number of migrant; SE = standard error; nd = no determined; ns = no significance.

*P < 0.05, ***P < 0.0001.

### Genetic diversity

At the population level, the PPL was high, an average of 94.4 %, ranging from 77.8 % in Phuoc Thuan to 100 % in five populations of Ham Minh, Tan Thuan, Bung Rieng, Binh Chau and Bong Trang. The mean number of alleles per locus (*N*_A_) in each population was 2.4. The Bung Rieng population had the highest *N*_A_ value (2.8) and Tan Ha had the lowest *N*_A_ values (2.0). The effective allele (*A*_E_) varied from 1.3 in Ham Minh to 1.9 in Bung Rieng, with an average of 1.6. The private alleles (*N*_P_) were found in two populations of Tan Thuan and Bong Trang (Table 2). The observed heterozygosity (*H*_O_) value averaged 0.298, ranging from 0.18 in Ham Minh to 0.425 in Bung Rieng. The mean expected heterozygosity (*H*_E_) value was 0.324, ranging from 0.202 (Ham Minh) to 0.442 (Bung Rieng). Thus, Ham Minh had the lowest observed and expected heterozygosity, whereas the highest values were in Bung Rieng. The fixation index (*F*_IS_) varied from 0.053 (Binh Chau) to 0.166 (Cam Duc), with an average of 0.109, indicating an excess of homozygotes and inbreeding in all populations. The *F*_IS_ values were significantly positive in three populations of Ham Minh, Cam Duc and Bong Trang. The inbreeding corrected for null alleles based on the individual inbreeding model (*F*_IS_IIM) varied from 0.02 in Phuoc Thuan to 0.085 in Tan Ha, with an average of 0.031, also indicating homozygote excess. However, this value was low compared to the fixation index *F*_IS_. BOTTLENECK analysis showed that a significant heterozygosity deficit was detected in Ham Minh and Bung Rieng. A significant heterozygosity excess was only found in Bung Rieng. This suggests that there is evidence of a recent bottleneck in some studied populations.

**Table 2.** Genetic diversity values and results of bottleneck tests for eight *D. condorensis* populations.

Populations	*N*	*P* (%)	*N*_A_	*A*_E_	*N*_P_	*H*_O_ (SE)	*H*_E_ (SE)	*F*_IS_ (SE)	*F*_IS_IIM	*P-*value of bottleneck		
										A	B	C
Ham Minh	21	100	2.3	1.3	—	0.18	0.202	0.133*	0.054	0.007	ns	0.01
Cam Duc	25	88.9	2.3	1.6	—	0.267	0.312	0.166**	0.067	ns	ns	ns
Tan Ha	25	88.9	2	1.4	—	0.253	0.269	0.092	0.085	ns	ns	ns
Tan Thuan	23	100	2.7	1.7	1	0.304	0.348	0.148	0.041	ns	ns	ns
Phuoc Thuan	19	77.8	2.3	1.6	—	0.316	0.327	0.078	0.02	ns	ns	ns
Binh Chau	21	100	2.4	1.5	—	0.291	0.3	0.053	0.064	ns	ns	ns
Bung Rieng	23	100	2.8	1.9	—	0.420	0.445	0.061	0.022	ns	0.018	0.037
Bong Trang	26	100	2.7	1.8	1	0.35	0.393	0.146*	0.024	ns	ns	ns
Mean		94.4	2.4	1.6		0.298 (0.021)	0.324 (0.022)	0.109	0.031			

*N* = sample size; *P* = percentage of polymorphic loci; *N*_A_ = alleles per locus; *A*_E_ = effective alleles; *N*_P_ = number of private alleles; *H*_O_ and *H*_E_ = observed and expected heterozygosities; *F*_IS_ = fixation index; *F*_IS_IIM = corrected inbreeding coefficient for null alleles; A = heterozygosity deficit, one-tailed test; B = heterozygosity excess, one-tailed test; C = heterozygosity excess or deficit, two-tailed test; SE = standard error.

*P < 0.05, **P < 0.01.

### Population genetic differentiation

Genetic differentiation (*F*_ST_) over all *D. condorensis* populations for each locus varied from 0.046 (dipt8) to 0.291 (dipt7) with an average of 0.122, indicating relatively low differentiation (Table 1). Furthermore, the pairwise *F*_ST_ values between populations ranged from 0.024 to 0.138 **[see****Supporting Information—Table S4****]**. The highest *F*_ST_ value appeared between Ham Minh and Phuoc Thuan (0.138), whereas the lowest appeared between Ham Minh and Cam Duc (0.024). Significant genetic differentiations were found between populations (*P* < 0.05), except the *F*_ST_ value between Ham Minh and Binh Chau. The average number of migrants (*N*_m_) per generation was 2.589, which was considered high. The lowest *N*_m_ value was found at dipt7 (*N*_m_ = 0.609) and the highest value was at dipt8 (*N*_m_ = 5.132). Analysis of molecular variance was performed to investigate genetic variations among and within populations on the basis of 10 000 permutations. The results showed that 13.36 % of the total genetic variation occurred among populations, and 86.64 % of the population variation was related to the heterozygosity of individuals within populations **[see****Supporting Information—Table S5****]**. The variation among populations was significant (*P* < 0.0001).

### Genetic structure

**Figure 1.**
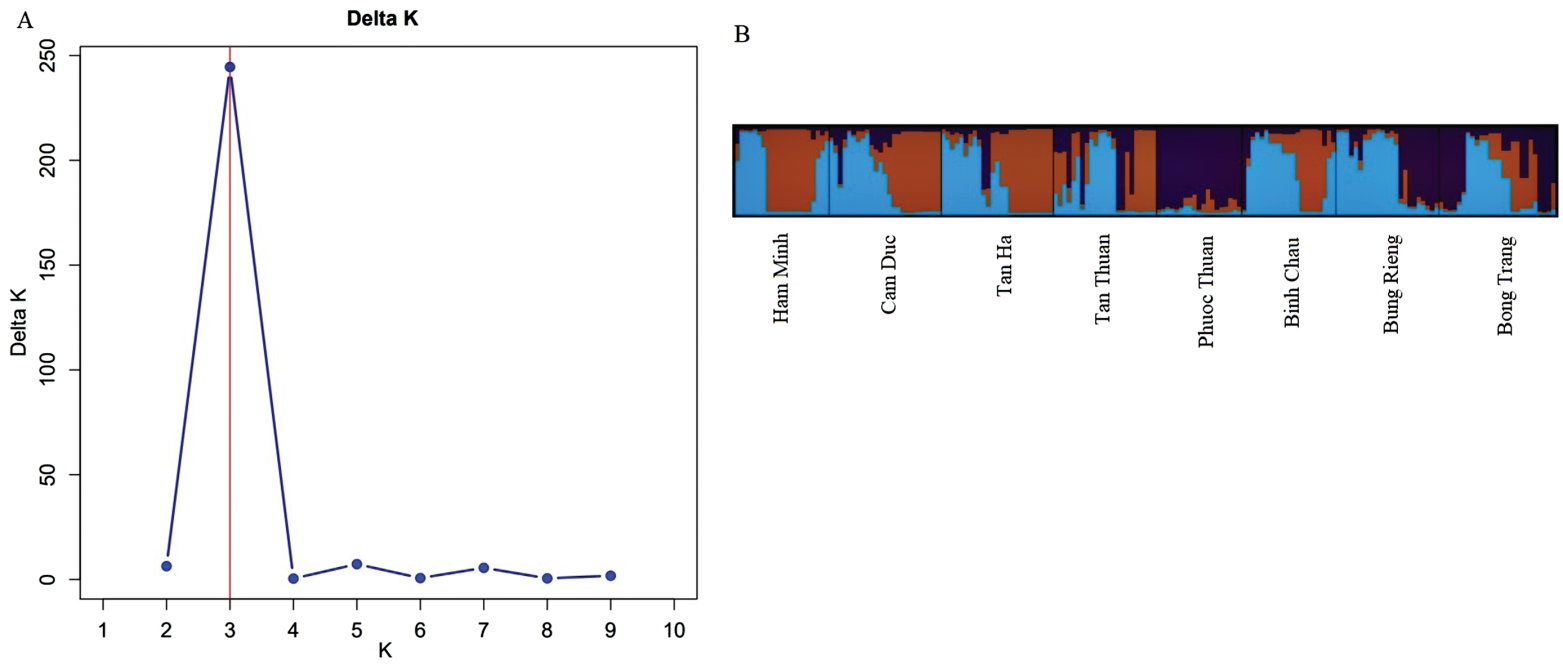
Results of STRUCTURE analysis for the eight *D. condorensis* populations. (A) Delta*K* with cluster number *K* from 1 to 10. (B) Barplot of admixture assignment for the 183 individuals of eight populations with *K* = 3.

**Figure 2.**
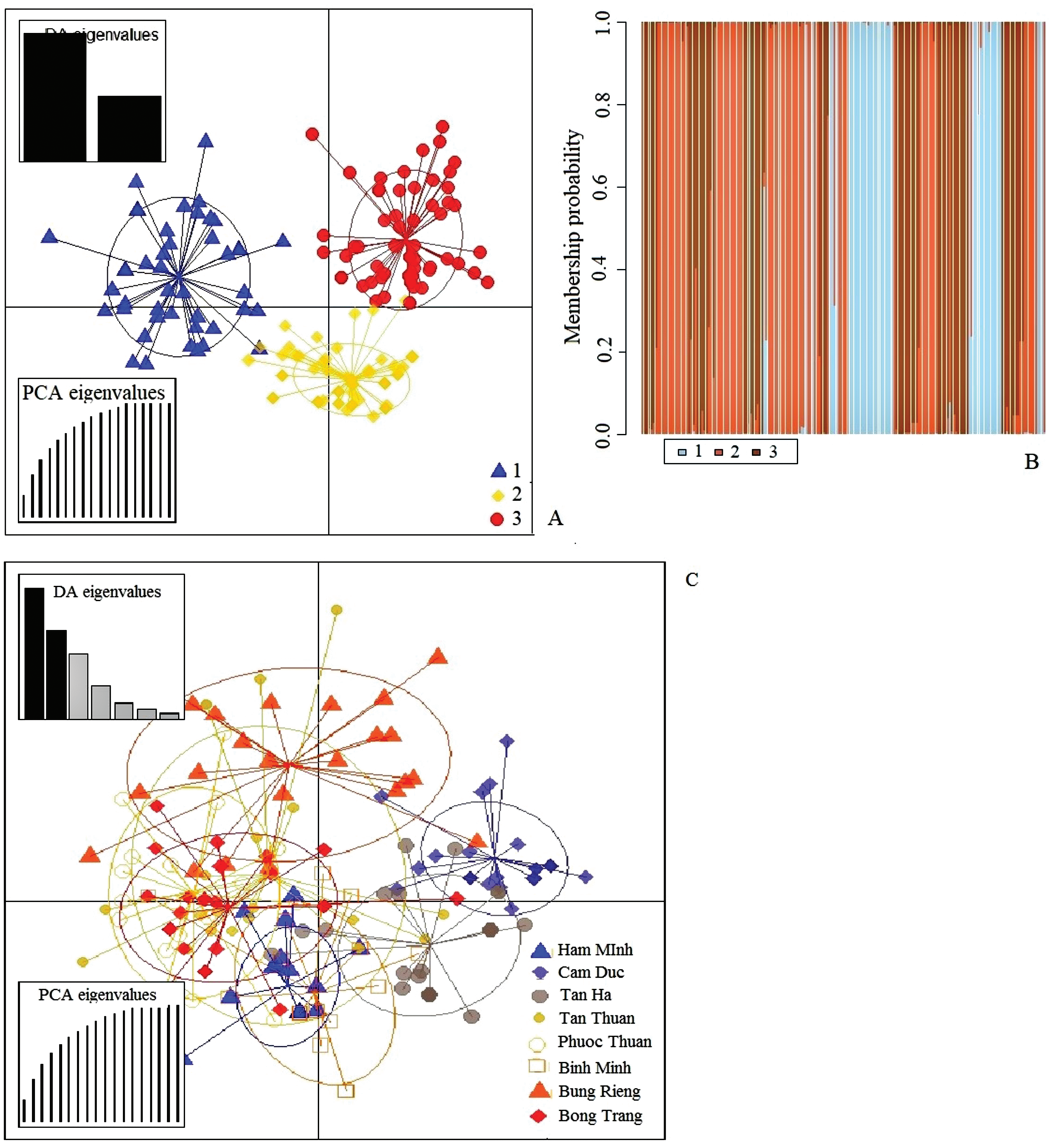
Analysis of population structure using DAPC. (A) Barplot shows assignment of individuals to the three genetic clusters without prior information. (B) Scatterplot of the DAPC without prior information. (C) Scatterplot of the DAPC with prior information.

We evaluated the population structure of the 183 individuals of *D. condorensis* using an admixture model based on the Bayesian analysis. The results showed that the optimum number of genetic groups (*K*) was three, with the highest value of delta *K* (244.5) obtained from STRUCTURE HARVESTER (Fig. 1A). At *K* = 3, the bar plot of admixture assignment for each individual was exhibited in Fig. 1B. The proportion of colour of 183 *D. condorensis* individuals showed the fraction of ancestry associated with each genetic group. Individuals with a proportion of ancestry value higher than 80 % were considered as no admixture individuals, and values less than 80 % as admixture individuals. The results indicated that the blue group was composed of 68 individuals with 39 no admixture and 19 mixture individuals, whereas the green group consisted of 57 individuals with 40 no admixture and 17 mixture individuals. The red group consisted of 58 individuals with 39 no admixture and 19 admixture individuals. However, only the Phuoc Thuan population included entirely individuals from the red group, and the other populations included individuals from three groups. Discriminant analysis of principal components without prior information revealed three genetic clusters, after 16 PCs were retained using the *xval*DAPC function (Fig. 2A and B). The first discriminant function separated three clusters clearly **[see****Supporting Information—Fig. S2****]**. All individuals from Phuoc Thuan and most of individuals from Bong Trang were assigned to the cluster 1. Some individuals from the five populations of Tan Ha, Tan Thuan, Bung Rieng and Binh Chau were also assigned to this cluster, except individuals from two populations of Ham Minh and Cam Duc **[see****Supporting Information—Table S6****and****Figs S3****and****S4****]**. The second cluster was composed of most of individuals from four populations of Ham Minh, Cam Duc, Tan Ha and Tan Thuan. Several individuals from three populations of Bung Rieng, Binh Chau and Bong Trang were assigned to the second cluster, while the third cluster was composed of most of individuals from two remaining populations of Bung Rieng and Binh Chau. A few individuals from four populations of Ham Minh, Cam Duc, Tan Ha and Tan Thuan were also assigned to the third cluster. The DAPC with prior information found individuals within and among *D. condorensis* populations (Fig. 2C). The high overlap of genetic clusters indicated a low degree of differentiation among populations. The results from this analysis identified that high overlap was found between Ham Minh and Binh Chau (*F*_ST_ = 0.024), and between Bong Trang and Phuoc Thuan (*F*_ST_ = 0.029), whereas Bung Rieng was separated from two populations of Bong Trang and Phuoc Thuan. Tan Thuan was differentiated from Tan Ha and Cam Duc. However, Tan Thuan was closer to Bong Trang than Cam Duc and Tan Ha.

## Discussion

Genetic diversity in species is often associated with geographic distribution range, population size, longevity, mating system, migration and balancing selection (White *et al.* 2007). High levels of genetic diversity reflect better adaptations of species to the environment (Hamrick and Godt 1996; Dick *et al.* 2008). In the present study, we evaluated the genetic diversity and population structure of *Dipterocarpus condorensis* to provide essential information for conservation actions needed in the future. This is an endangered species with a narrow geographic distribution and small population size; therefore, the genetic diversity is expected to be low compared to species with a wide distribution range and large population size (Hamrick and Godt 1996). Prior to this study, little information was found in public databases on genetic diversity within and among *D. condorensis* populations in lowland tropical forests. Previous studies mainly focused on morphological and ecological characteristics (Nghia 2005; Bien and Huong 2011). In our study, *D. condorensis* maintained relatively low genetic diversity (*N*_A_ = 2.4, *H*_O_ = 0.296 and *H*_E_ = 0.324) compared with previously published studies for some other species, such as *S. leprosula* (Ng *et al.* 2004), *S. robusta* (Pandey and Geburek 2009), *D. aromatica* (Harada *et al.* 2018), and *D. dyeri* (Tam *et al.* 2020). However, our results showed a similar level of genetic diversity found previously in several studies, *P. malaanonan* (Abasolo *et al.* 2009), *Shorea javanica* (Rachmat *et al.* 2012), *D. alatus* (Tam *et al.* 2014) and *H. odorata* (Trang *et al.* 2014). These results indicated that low genetic diversity level was strongly related to anthropogenic disturbance. Deforestation and over-exploitation might be the major factors contributing to the decrease in genetic diversity in all studied populations. In addition, *D. condorensis*, an endemic species is restricted due to its distribution range and isolation in Vietnam. Genetic diversity within populations is associated with the degree of anthropogenic disturbances (Honnay and Jacquemyn 2007), can be decreased through genetic drift and increased homozygosity for common alleles due to the loss of rare alleles (Gijbels *et al.* 2015). This suggested that populations are inherently vulnerable and then at a low risk of extinction (Falk and Holsinger 1991). Among the five seriously disturbed populations, four populations of Cam Duc, Ham Minh, Tan Ha and Binh Chau had low genetic diversity, with the exception of Phuoc Thuan. Of the three preserved populations in the nature reserves, two populations of Bung Rieng and Bong Trang maintained higher genetic diversities, while the Tan Thuan population had lower genetic diversities. These suggest that past habitat disturbances may have negatively affected the genetic diversity of *D. condorensis* populations. The studied populations might be suffered from disturbance, although some populations are protected in nature reserves. The present populations are fragmented into subpopulations with few individuals. Fragmented habitats leading to increased isolation between populations might have reduced gene exchange among populations via both pollen and seeds in recent decades. This is consistent with the suggestion that low genetic diversity is related to a disturbance degree and small population size. Significant mean heterozygote deficiency (*F*_IS_ = 0.109) across 183 wild trees from eight populations suggests that relatively high heterozygous deficiency may exist within the distribution range of *D. condorensis*. This suggested the existence of inbreeding in small populations, although the dipterocarp is the predominantly outcrossing species (Appanah and Chan 1981; Ashton 1982). Their flowers are pollinated by insects and the fruits are dispersed by wind and water currents. We also identified heterozygous deficiency of *D. condorensis* by BOTTLENECK analysis and showed a reduction in population size of the Ham Minh and Bung Rieng populations. This suggests that the change in population size has occurred over the past few decades.

Our results showed a relatively low genetic differentiation among populations with the *F*_ST_ value of 0.122 and the *G*ʹ_ST_ value of 0.149 and reflected relatively high level of migration among *D. condorensis* populations (*N*_m_ = 2.585). Similarly, our AMOVA analysis showed low significant variation among *D. condorensis* populations (*P* < 0.0001). In previous studies, similar results were reported low genetic differentiation and reflected the high level of gene flow, such as *Shorea lumtensis* (Lee *et al.* 2006); *S. javanica* (Rachmat *et al.* 2012; Muhammad *et al.* 2016); *D. dyeri* (Tam *et al.* 2019). This is consistent with large longevity and predominately outcrossing species (Brutting *et al.* 2012) and seemed to be associated with a low genetic structure. Species with outcrossing have low genetic differentiation among populations (Momose *et al.* 1994; Hamrick and Godt 1996; Finkeldey and Hattemer 2007; Kettle *et al.* 2011). Population genetic differentiation is strongly affected by gene flow and genetic drift (Schaal *et al.* 1998; De Morais *et al.* 2015). Genetic differentiation is affected by gene flow and genetic drift (Slatkin and Barton 1989). High gene flow (Nm > 1) showed that the number of migrants per generation inferred from nine loci. In the present study, relatively high gene flow could counteract the effects of genetic drift, reducing genetic differentiations among populations, whereas increasing genetic variation within the populations. As a perennial tree, *D. condorensis* is pollinated by insects (bees) and its seeds are dispersed by wind and small mammals (squirrels and bats). The dispersal of pollen grains is mainly depended on insects. Therefore, increasing isolation distance will reduce pollen transmission among populations. Fragmentation can affect the genetic structure of this species. The lowest genetic differentiation (0.024) was found between populations Ham Minh and Binh Chau related to the closest geographic distance. The highest genetic differentiation (0.138) was found between Ham Minh and Phuoc populations, having the longest geographic distance. These suggest that geographic distance may restrict gene flow between these populations. However, no similar findings were found between Phuoc Thuan and Bung Rieng (*F*_ST_ = 0.109), Phuoc Thuan and Cam Duc (*F*_ST_ = 0.1). The analyses of STRUCTURE and DAPC without prior information revealed three major genetic groups for 183 wild *D. condorensis* trees. One genetic group was composed of two populations Phuoc Than and Bong Trang. The second group was composed of four populations Cam Duc, Ham Minh, Tan Ha and Tan Thuan. The third group included two remaining populations Bung Rieng and Binh Chau. However, the considerable mixing of the three groups in several populations was determined, suggesting that genetic differentiation was relatively low and gene flow was high among these populations. This can also be found in the DAPC analysis with prior information. The mixing of the three groups was found in five populations Tan Ha, Tan Thuan, Bung Rieng, Binh Chau and Bong Trang, while the mixing of the two groups was observed in Cam Duc and Ham Minh populations. No admixture was found in the Phuoc Thuan population. These isolated populations could be related to anthropogenic disturbance. A reduction in the dispersal of pollen grains and seeds could be considered as a barrier to gene flow for *D. condorensis* species.

## Conclusions

In the present study, we investigated the genetic diversity and genetic structure of *D. condorensis* based on a sampling strategy involving its natural distribution areas, and determined the low level of overall genetic diversity and the genetic diversity of its populations. Some populations might have suffered from anthropogenic disturbance. The STRUCTURE analysis showed that three genetic groups were consistent with the DAPC with a prior information. Genetic variation mainly existed within *D. condorensis* populations. Furthermore, private alleles were found in Tan Thuan and Bong Trang populations. These are resources that contribute to the maintenance and evolution of species. Therefore, in developing a conservation strategy for *D. condorensis*, populations with high genetic diversity and private alleles, such as Bong Trang, Bung Rieng and Tan Thuan should focus on *in situ* conservation, meanwhile the remaining populations should be collected for *ex situ* conservation as a germplasm source of the species in the future.

## Supporting Information

The following additional information is available in the online version of this article—

Table S1. Collection localities of *Dipterocarpus condorensis.*

Table S2. Nucleotide sequences of the SSR primers, allele size range, genetic diversities for *Dipterocarpus condorensis.*

Table S3. Microsatellite (SSR) raw data for *Dipterocarpus condorensis*.

Table S4. Pairwise genetic differentiation (*F*_ST_) between the eight *Dipterocarpus condorensis* populations.

Table S5. Analysis of molecular variance from eight *Dipterocarpus condorensis* populations.

Table S6. Number of individuals for each population assigned to a cluster.

Figure S1. Location of the study sites and habitats of *Dipterocarpus condorensis*.

Figure S2. Densities of individuals on the first discriminant function. Each colour shows one genetic cluster using DAPC without prior information.

Figure S3. Number of individuals for each population (rows) assigned to each of the three inferred genetic clusters (columns) using the DAPC without prior information.

Figure S4. Individuals (rows) assigned to the genetic clusters (columns) based on discriminant functions. Colour shows membership probabilities to each genetic cluster (red = 1, orange = 0.75, yellow = 0.25 and white = 0 and blue crosses show the cluster using DAPC).

plac007_suppl_Supplementary_Material

plac007_suppl_Supplementary_Table_S3

## Data Availability

The raw data are available in **Supporting Information—Table S3**.

## Notes

**Natural History and Conservation. Chief Editor**: F. Xavier PicÃ³
