# Supplementary material for: Genetic structure of the endemic Dipterocarpus condorensis revealed by microsatellite markers
Source: AoB Plants. 2022 Feb 23;14(2):plac007. doi: 10.1093/aobpla/plac007 (PMC9011383; doi:10.1093/aobpla/plac007)
Supplement: plac007_suppl_Supplementary_Material [file plac007_suppl_supplementary_material.pdf]

Genetic structure of the endemic *Dipterocarpus condorensis* Pierre revealed by microsatellite markers

Table S1. Collection localities of *Dipterocarpus condorensis*

Population	Sampling size	Collection locality	Altitude	Latitude	Longitude
Ham Minh	25	Ham Thuan Nam, Binh Thuan	92-117 m	10°49'N	107°54'E
Cam Duc	25	Cam Lam, Khanh Hoa	121-136 m	12°03'N	109°09'E
Tan Ha	23	Ham Tan, Binh Thuan	118-138	10°45'N	107°35'E
Tan Thuan	21	La Gi, Binh Thuan	65-87 m	10°48'N	107°54'E
Phuoc Thuan	23	Xuyen Moc, Ba Ria - Vung Tau	96-125 m	10°29'N	107°25'E
Binh Chau	26	Xuyen Moc, Ba Ria – Vung Tau	87-110 m	10°32'N	107°31'E
Bung Rieng	19	Xuyen Moc, Ba Ria – Vung Tau	95-112 m	10°31'N	107°30'E
Bong Trang	21	Xuyen Moc, Ba Ria – Vung Tau	104-120 m	10°28'N	107°26'E

Table S2. Nucleotide sequences of the SSR primers, allele size range for *D. condorensis*.

Locus	Nucleotide sequences (5'-3')	Repeat motif	T_m (^oC)	PCR product length (bp)
dipt1[1]	F: 5'-CTTCCCTAAATTCCCCAATGTT-3'<br>R: 5'-TAATGGTGTGTGTACCAGGCAT	$(AG)_{15}$	55	193-211
dipt2[1]	F: 5'-ACAATGAAACTTGACCACCCAT-3'<br>R: 5'-CAAAAGGACATACCAGCCTAGC-3'	$(GA)_{24}$	56	228-240
dip3[1]	F: 5'-TAGGGCATATTGCTTTCTCATC-3'<br>R: 5'-CTTATTGCAGTCATCAAGGGAA-3'	$(AG)_{15}$	55	214-226
dipt4[1]	F: 5'-TCTCAAAATCTGCAAAGACAGC-3'<br>R: 5'-CCATAGTCATCACCTCTAATGGTC-3'	(GA)25	55	241-301
dipt5[1]	F: 5'-TGGCAAACAAGCTACTGTTCAT-3'<br>R: 5'-CATGGGTTTAGCAACCTACACA-3'	$(TA)_8$	56	258-262
dipt6[1]	F:5'- CAGGAGGGGAATATGGAAAA-3'<br>R: 5'-AAGTCGTCATCTTTGGATTGC-3'	$(AC)_9$	54	122-140
dipt7[2]	F: ATGCTTACCACCAATGTGAATG-3'<br>R: CTCGCAGCAGAACAACTTTCTA-3'	$(GA)_6$	55	170-180
dipt8[3]	F: 5'-ATCTGTTCTTCTACAAGCC-3'<br>R: 5'-TTAGAACTTGAGTCAGATC-3'	(CT)4TT(CT)5	54	166-178
dipt9[3]	F: 5'-ATGTC CATGT TTGAG TG-3'<br>R: 5'-CATGG ACATA AGTGG AG-3'	$(CT)_8CA(CT)_5$ CACCC$(CTCA)_3CT(CA)_{10}$	55	170-180

Notes: F, forward primer; R, reverse primer; T_m, PCR annealing temperature

[1]Isagi V, Kenta T, Nakashizuka T. 2002. Microsatellite loci for a tropical emergent tree, *Dipterocarpus tempehes* V. S1 (Dipterocarpaceae). Molecular Ecology Notes **2**:12-13.

[2]Terauchi R. 1994. A polymorphic microsatellite marker from the tropical tree *Dryobalanops lanceolata* (Dipterocarpaceae). Japan Journal of Genetics **69**:567-576.

[3]Ujino T, Kawaharam T, Tsumara Y, Nagamitsu T, Yoshimaru H, Ratnam W. 1998. Development and polymorphism of simple sequence repeat DNA markers for *Shorea curtisii* and other Dipterocarpaceae species. Heredity **81**:422-428.

Table S4. Pairwise genetic differentiation (F_{ST}) between the eight *D. condorensis* populations using GenAlEx[1].

Population	Ham Minh	Cam Duc	Tan Ha	Tan Thuan	Phuoc Thuan	Binh Chau	Bung Rieng
Cam Duc	0.091						
Tan Ha	0.084	0.045					
Tan Thuan	0.054	0.055	0.043				
Phuoc Thuan	0.138	0.098	0.11	0.074			
Binh Chau	0.024	0.076	0.07	0.058	0.1		
Bung Rieng	0.094	0.075	0.096	0.047	0.109	0.076	
Bong Trang	0.073	0.058	0.059	0.034	0.029	0.041	0.056

[1]Peakall R, Smouse PE. 2012. GenAlEx 6.5: genetic analysis in excel. Population genetic software for teaching and research an update. *Bioinformatics* **28**:2537-2539.

Table S5. Analysis of molecular variance from eight *D. condorensis* populations using Arlequin v.3.5[1].

	df*	Sum of squares	Variance components	Total variation (%)	P value
Among populations	7	84.5	0.231	13.36	<0.0001
Within populations	358	537.2	1.5	86.64	
Total	365	621.7	1.732		

[1]Excoffer L, Laval G, Schneider S. 2005. Arlequin v. 3.5. an integrated software package for population genetics data analysis. *Evolutionary Bioinformatics online* **1**:47-50.

Table S6. Number of individuals for each population assigned to a cluster using DAPC[1]

Population	Cluster 1	Cluster 2	Cluster 3
Ham Minh	-	15	6
Cam Duc	-	14	11
Tan Ha	2	14	9
Tan Thuan	5	15	3
Phuoc Thuan	19	-	-
Binh Chau	1	9	11
Bung Rieng	8	2	13
Bong Trang	10	7	9

[1]Jombart T, Devillard S, Balloux F. 2010. Discriminant analysis of principal components: a new method for the analysis of genetically structured populations. *BMC Genetics* **11**:94.

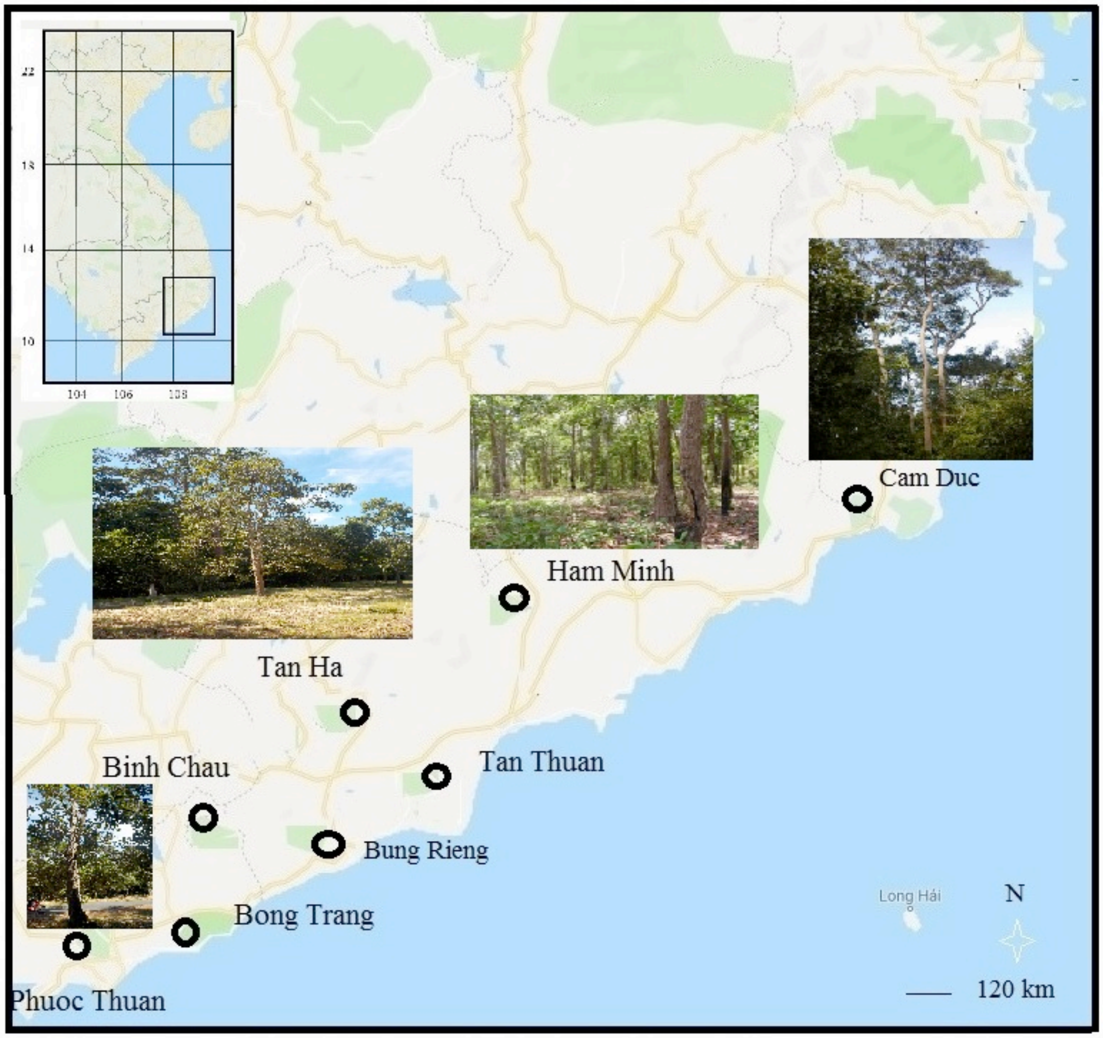

Figure S1. Location of the study sites and *Dipterocarpus condorensis* habitats.

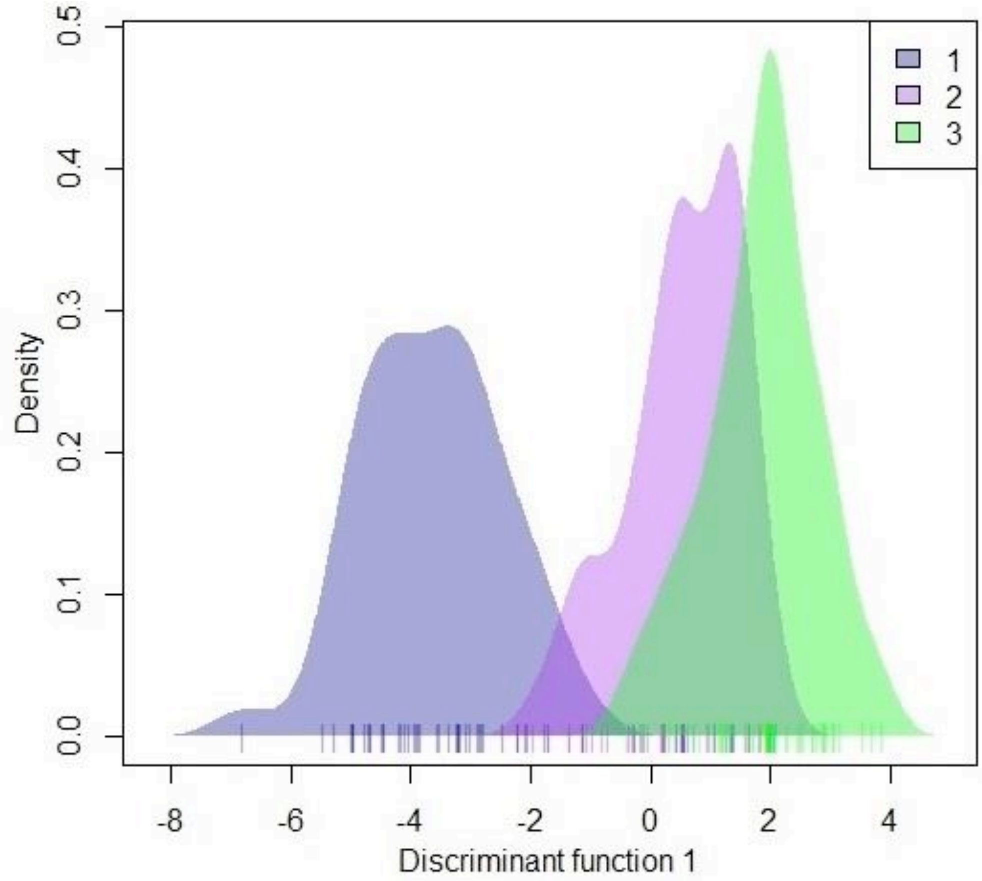

Figure S2. Densities of individuals on the first discriminant function. Each color shows one genetic cluster using DAPC without prior information.

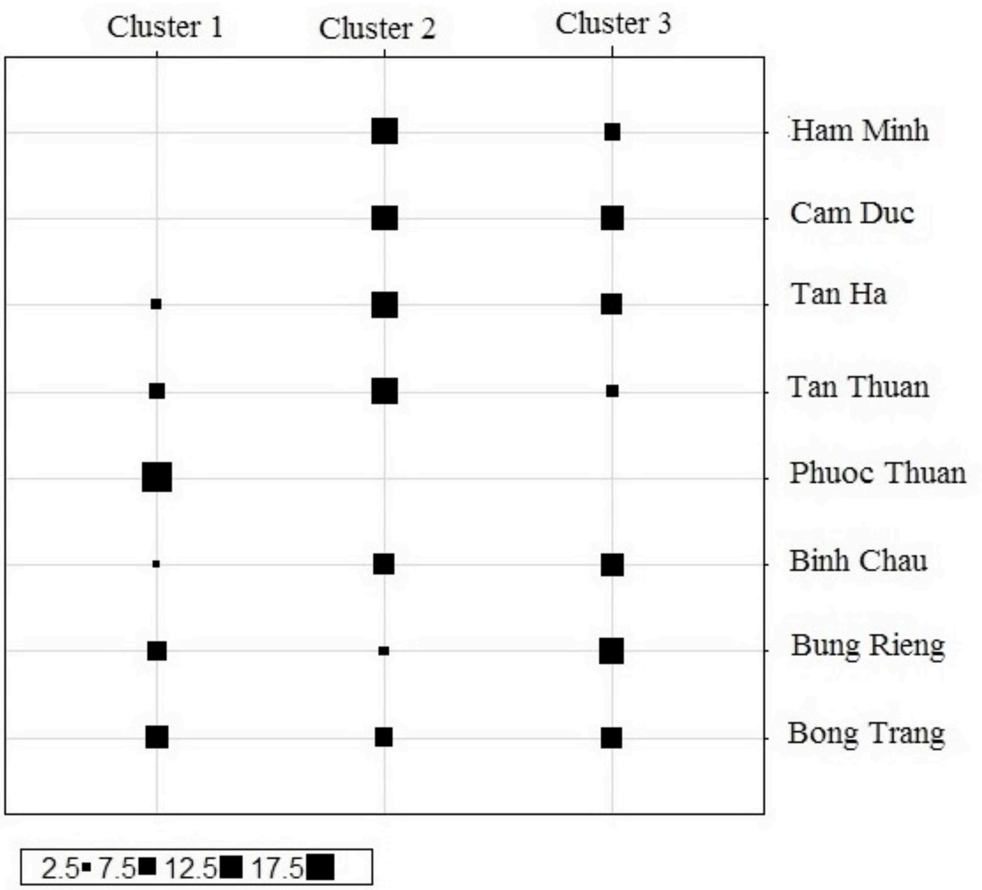

Figure S3. Number of individuals for each population (rows) assigned to each of the three inferred genetic clusters (columns) using the DAPC without prior information.

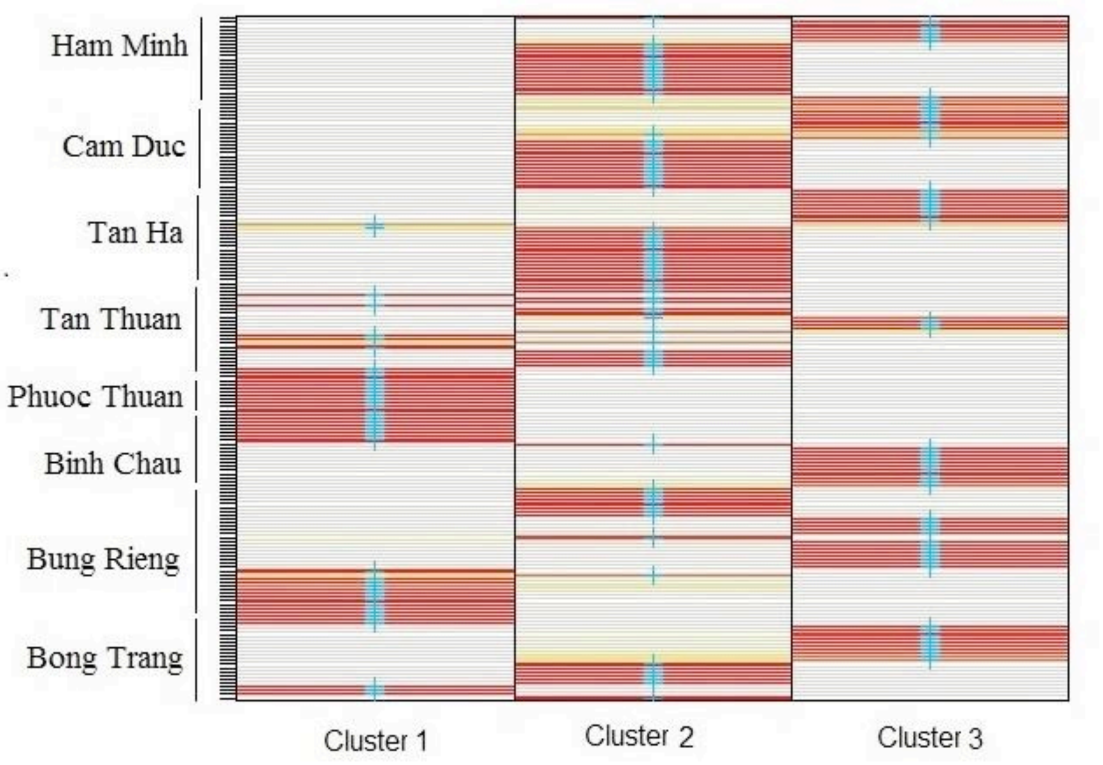

Figure S4. Individuals (rows) assigned to the genetic clusters (columns) based on discriminant functions. Color shows membership probabilities to each genetic cluster (red = 1, orange = 0.75, yellow = 0.25 and white = 0 and blue crosses show the cluster using DAPC.
